# Cough associated with the detection of *Mycoplasma hyopneumoniae* DNA in clinical and environmental specimens under controlled conditions

**DOI:** 10.1186/s40813-022-00249-y

**Published:** 2022-01-25

**Authors:** Ana Paula S. Poeta Silva, Gabriel Y. Storino, Franco S. Matias Ferreyra, Min Zhang, Eduardo Fano, Dale Polson, Chong Wang, Rachel J. Derscheid, Jeffrey J. Zimmerman, Maria J. Clavijo, Bailey L. Arruda

**Affiliations:** 1 Department of Veterinary Diagnostic and Production Animal Medicine, Iowa State University, 1920 Dayton Ave, Ames, IA 50010 USA; 2 School of Agricultural and Veterinarian Sciences, São Paulo State University (Unesp), Jaboticabal, SP Brazil; 3 Department of Statistics, College of Liberal Arts and Sciences, Iowa State University, Ames, IA USA; 4 Boehringer Ingelheim Animal Health US Inc., Atlanta, GA USA; 5 Pig Improvement Company, PIC®, Hendersonville, TN USA; 6 US Department of Agriculture, Agricultural Research Service, National Animal Disease Center, Ames, IA USA

**Keywords:** *Mycoplasma hyopneumoniae*, Enzootic pneumonia, Tracheal swabs, Oral fluids, Water samples, Air samples, Cough, Pathology

## Abstract

**Background:**

The association of cough with *Mycoplasma hyopneumoniae (MHP)* DNA detection in specimens was evaluated under conditions in which the *MHP* status of inoculated and contact-infected pen mates was closely monitored for 59 days post-inoculation (DPI).

**Methods:**

Seven-week-old pigs (n = 39) were allocated to five rooms (with one pen). Rooms contained 9 pigs each, with 1, 3, 6, or 9 *MHP*-inoculated pigs, respectively, except Room 5 (three sham-inoculated pigs). Cough data (2 × week) and specimens, tracheal swabs (2 × week), oral fluids (daily), drinker wipes (~ 1 × week), and air samples (3 × week) were collected. At 59 DPI, pigs were euthanized, and lung and trachea were evaluated for gross and microscopic lesions. Predictive cough value to *MHP* DNA detection in drinker and oral fluid samples were estimated using mixed logistic regression.

**Results:**

Following inoculation, *MHP* DNA was first detected in tracheal swabs from inoculated pigs (DPI 3), then oral fluids (DPI 8), air samples (DPI 10), and drinker wipes (21 DPI). *MHP* DNA was detected in oral fluids in 17 of 59 (Room 1) to 43 of 59 (Room 3) samples, drinker wipes in 4 of 8 (Rooms 2 and 3) to 5 of 8 (Rooms 1 and 4) samples, and air samples in 5 of 26 (Room 2) or 3 of 26 (Room 4) samples. Logistic regression showed that the frequency of coughing pigs in a pen was associated with the probability of *MHP* DNA detection in oral fluids (*P* < *0.01*) and nearly associated with drinker wipes (*P* = *0.08*). Pathology data revealed an association between the period when infection was first detected and the severity of gross lung lesions.

**Conclusions:**

Dry, non-productive coughs suggest the presence of *MHP*, but laboratory testing and *MHP* DNA detection is required for confirmation. Based on the data from this study, oral fluids and drinker wipes may provide a convenient alternative for *MHP* DNA detection at the pen level when cough is present. This information may help practitioners in specimen selection for *MHP* surveillance.

## Background

*Mycoplasma hyopneumoniae* (*MHP*) is an impactful health challenge in swine [1], resulting in reduced daily weight gain and poor feed conversion [2]. *MHP* binds to ciliated epithelial cells of the respiratory tract by means of adhesins [3, 4], resulting in ciliostasis and diminished function of the mucociliary apparatus [5]. *MHP* virulence factors, i.e., adhesins and lipid associated membrane proteins, elicit a pro-inflammatory response, leading to infiltration and accumulation of immune cells, i.e., lymphocytes, plasma cells, and neutrophils, in the lumen and interstitium surrounding the conducting airways [6–8]. As the infection becomes chronic, marked hyperplasia of the bronchus-associated lymphoid tissues (BALT) and well-demarcated cranioventral pulmonary consolidation are identified as histologic and gross lesions, respectively, and are characteristic of porcine enzootic pneumonia (PEP). Due to bronchoconstriction resulting from the obstruction of airways, extensive cough may be observed in *MHP*-infected pigs [9–11].

Effective control measures are dependent on an accurate understanding of the limitations and benefits of sample techniques and diagnostic assays to assess herd status within specific clinical context [12]. Several different sample techniques and diagnostic tests have been developed to detect *MHP* nucleic acid, antigen or antibody, and are available for surveillance programs [13]. Yet, the selection of samples hinges on the diagnostic objectives, the level of disease in the herd, accuracy of the test, and cost.

Serum antibody based on enzyme-linked immunosorbent assay (ELISA) is commonly used to monitor swine herds. However, the utility of serological assays for *MHP* can be hindered by the limited correlation between a positive assay and disease, inability to differentiate natural infection from vaccination, the highly variable time lapse between infection and antibody production, variable diagnostic accuracy across commercially available ELISAs, and antibody cross-reactions with other mycoplasmas [13, 14]. Consequently, it is difficult to interpret results based on the individual or at the herd level.

In contrast, polymerase chain reaction (PCR) using specimens of the respiratory tract can determine the direct presence of the *MHP* deoxyribonucleic acid (DNA). The highest concentration of *MHP* DNA has been described in bronchoalveolar lavage fluid (BALF) over the course of infection [15]. Yet, BALF sampling is most commonly performed at post mortem examination or in sedated animals [16]. Alternatively, tracheal and tracheobronchial swabs for the detection and recovery of *MHP* have been described as the preferred sample for early detection in live pigs [17–19], followed by laryngeal swabs [20]. However, collection of such samples can be laborious given that it requires a specific number of trained personnel (two or three) and is stressful for the animals because of intensive restraint [17–19].

Several swine pathogens are present in and/or transmitted through oral fluids. Pen-based oral fluid is a non-invasive, cost-effective, aggregated sampling method to detect a number of swine pathogens. Pathogen surveillance for porcine circovirus 2 (PCV2), porcine reproductive and respiratory syndrome virus (PRRSV), and influenza A virus (IAV) through the detection of specific viral genes or antibodies in oral fluids has been successful [21]. The use of oral fluids for *MHP* DNA detection may be precluded due to the lower sensitivity than tracheal and laryngeal samples under field conditions [17]. Yet, there is very limited information concerning the agreement of oral fluid-based PCR for the detection of *MHP* DNA and other diagnostic samples with the predominate clinical sign of PEP, coughing [17, 22]. Establishing the diagnostic parameters of different sampling techniques of *MHP* and correlation between the PCR results of different sample types and clinical signs is critical for the strategic implementation of disease prevention and elimination/control strategies that will mitigate the economic and production losses incurred due to PEP. Therefore, this study investigated the predictive value of coughing for the detection of *MHP* DNA in aggregated specimens, such as pen-based oral fluids, pen-based drinker wipes, and air samples collected from pigs housed in rooms differing in within-pen *MHP* prevalence.

## Materials and methods

### Experimental design

Seven-week-old *MHP*-naïve pigs (n = 39) were blocked by litter (n = 19) and randomly assigned to one of five rooms: (Room 1) one *MHP*-inoculated pig commingled with eight uninoculated pigs (n = 9), (Room 2) three *MHP*-inoculated pigs with six uninoculated pigs (n = 9), (Room 3) six *MHP*-inoculated pigs with three uninoculated pigs (n = 9), (Room 4) nine *MHP*-inoculated pigs (n = 9), and (Room 5) 3 negative control pigs (n = 3). Clinical data collected, samples tested for *MHP* PCR, and pathologic evaluations are presented in Table 1. Twice weekly, coughing was assessed by individual pig and room by an observer blinded to inoculation status, and digital recording system captured cough daily. *MHP* DNA was tested by Real-Time qPCR testing in deep tracheal swabs, pen-based oral fluid, pen drinker, and pen air samples. On day post-inoculation (DPI) 59, pigs were humanely euthanized, and lung tissue samples were collected for post mortem examination. All procedures were conducted with the approval of the Iowa State University Office for Responsible Research and Institutional Animal Care and Use Committee.

**Table 1:** Clinical data collected, samples tested for *Mycoplasma hyopneumoniae* (*MHP*) PCR, and pathologic evaluations throughout the study

Samples	Frequency	Level^a^	No. of possible samples	No. of collected samples	Testing	Outcome
*Coughs*						
Over 27-min	2 × week	Individual	702	621	Blind observer^b^	Total coughs per pig (or per group)
Over 24-h	Real-time	Room	355	303	Digital recording system^c^	Total coughs per group
*Lung lesions*						
Gross	Necropsy	Individual	39	37	Blind observer^b^	% lung affected
Microscopic	Necropsy	Individual	39	39	Histopathology^b^	Score 0, 1, 2, 3, 4
*Tracheal swab*	2 × week	Individual	507	489	PCR Protocol 1^d^	*MHP* DNA Pos or neg; Ct value
*Oral fluid*	Daily	Rooms (1–5)	322	322	PCR Protocol 1	*MHP* DNA Pos or neg; Ct value
*Drinker*	1 × week	Room (1–5)	45	45	PCR Protocol 1	*MHP* DNA Pos or neg; Ct value
*Air*	3 × week	Room (2, 4, and 5)	84	84	PCR Protocol 2^e,f^	*MHP* DNA Pos or neg; Ct value

^a^Room 1: One *MHP*-inoculated pig commingled with 8 uninoculated pigs. Room 2: 3 *MHP*-inoculated pigs with 6 uninoculated pigs. Room 3: 6 *MHP*-inoculated pigs with 3 uninoculated pigs. Room 4: 9 *MHP*-inoculated pigs. Room 5: 3 uninoculated pigs

^b^Observer blinded to the *MHP* inoculation pig status

^c^Total number of coughs per day identified using a commercial digital quantitative cough recording system and software (SoundTalk®, SoundTalks NV, Leuven, Belgium)

^d^PCR Protocol 1: TaqMan® Fast Virus 1-Step Master Mix (Life Technologies, Carlsbad, CA USA) with AmpliTaq® 360DNA Polymerase (5U/uL) (Thermo Fisher Scientific, Inc., Waltham, MA USA)

^e^PCR Protocol 2: TaqMan® Fast Virus 1-Step Master Mix (Life Technologies)

^f^Collected from Room 2, 4, and 5

### Animals, housing and groups

Seven-week-old *MHP*-, IAV-, and PRRSV-negative pigs (n = 39) were received into a BSL-2 livestock infectious disease isolation facility accredited by the Association for Assessment and Accreditation of Laboratory Animal Care. At DPI -5, pigs were blocked by litter (n = 19) and randomly assigned (Microsoft Excel® random function) to one of five rooms: 9 pigs each in rooms 1 through 4 plus 3 pigs in the negative control room. Each room containing one pen was equipped with a single-pass non-recirculating ventilation system to prevent inadvertent aerosol transmission. On DPI -3, deep tracheal swabs were collected from all pigs and tested for *MHP* DNA (Real-Time qPCR). On DPI 0, after confirming all pigs to be negative for *MHP* infection, one randomly selected pig from Room 1, three pigs from Room 2, six pigs from Room 3, and nine pigs from Room 4 were intratracheally administrated a lung tissue homogenate (10 ml) containing *MHP* strain 232 [23] at concentration of ~ 1 × 10^5^ color-changing units per ml (Lot 45, Iowa State University Veterinary Diagnostic Laboratory, ISU VDL). Crude lung homogenate was originally prepared in a specific-pathogen-free pig by inoculating it with *MHP* strain 11 [24] which was isolated at Iowa State University in the 1960s. The isolate from this pig, identified as *MHP* strain 232, has been serially passaged in specific-pathogen-free pigs for production of crude lung homogenates. The resultant pneumonic lungs have been harvested at 4 weeks post-inoculation. This inoculum is efficient in reproducing *MHP* infection under controlled and field conditions [14, 17, 19, 20, 25, 26]. All negative control pigs were administrated Friis broth (10 ml; ISU VDL Doc 9.6726). Intratracheal inoculation was done using a feeding tube catheter (Integral Funnel, Two Eyes, Rounded Closed Tip, 4.7 mm × 41 cm, COVIDEN™ Kendall™, Coviden, Mansfield, MA USA) introduced past the larynx. Thereafter, animals were observed for 59 days.

### Sample collection

Deep tracheal swabs were taken at -3, 3, 7, 10, 14, 21, 24, 28, 35, 38, 45, 52, 59 DPIs from all pigs (Table 1). After restraining pigs with a snare and an oral speculum, a single-use artificial insemination catheter (IMV PCAI catheter, Nasco, Fort Atkinson, WI USA) was introduced into the distal part of the trachea and then a swab (100 mm, FLOQSwabs® 519CS01, Copan Diagnostics, Inc. Murrieta, CA USA) attached to the flexible inner tube was extended to collect mucus lining the trachea. To avoid contamination, the swab was retracted into the catheter before withdrawal from the larynx. After removal from the pig, the swab containing the collected material was severed from the inner rod and placed in a tube containing 1 mL sterile PBS. Pigs exhibiting signs of stress were administered a combination of tiletamine hydrochloride and zolazepam hydrochloride (500 mg; Telazol®, Zoetis Inc., Parsippany, NJ USA), xylazine (250 mg; XylaMed™, VetOne®, Boise, ID USA), and ketamine (250 mg; Zetamine™ Injection, VetOne®) at a dose of 1 mL per 4.4 kg of body weight prior to sample collection.

Aggregated samples included pen oral fluid samples, drinker wipe samples, and air samples. Pen-based oral fluid samples were collected as described elsewhere [21]. Beginning at DPI -4, pigs were provided daily access (25 min) to a 3-strand (1.6 cm diameter), 100% cotton rope (Web Rigging Supply, Lake Barrington, IL USA) suspended from a bracket fixed to the side of the pen (Table 1). Oral fluids were recovered by placing the wet portion of the rope inside a plastic bag and then passing the bag containing the wet rope through a wringer to express the fluid. Drinker wipe samples were collected on DPIs 0, 7, 14, 21, 24, 35, 45, 52, and 59 by wiping the surface (drinking nipple and water cup) with a paper towel and then recovering the absorbed liquid from the towel (Table 1). Air samples were collected three times per week in Rooms 2, 4, and 5 (negative control) by suspending an air sampler (Innovaprep prototype air collector, 200 L per min flow, Innovaprep, Drexel, MO USA) at ~ 25 cm above the pigs' heads in the center of the pen (Table 1). After a 60-min sampling period, collected air samples were eluted from the filter using 0.075% Tween 20/ PBS wet-foam (Innovaprep). Given the number of available air samplers for this study (n = 3), three rooms were selected to investigate the performance of the device in terms of specificity (Room 5, negative control pen) and in rooms highly *MHP*-infected (Room 4, e.g., with all 9 *MHP*-inoculated pigs) or undergoing transmission (Room 2, e.g., three *MHP*-inoculated pigs commingled with six uninoculated pigs). Following collection, all specimens were vortexed, transferred to 2 mL cryogenic tubes (Cryo.s™, Greiner Bio-One™), and then stored at −200980 °C until tested for *MHP* DNA.

### Assays

*MHP* DNA was tested by Real-Time qPCR testing in individual pig deep tracheal swabs, pen oral fluids, drinker, and air samples. Nucleic acid extraction was done using the MagMAX™-96 Pathogen RNA/DNA kit (Applied Biosystems™, Carlsbad, CA USA) and the Kingfisher™ Flex Purification System (Thermo Fisher Scientific, Inc., Waltham, MA USA). Tracheal swabs, pen-based oral fluids, and drinker samples were tested using TaqMan® Fast Virus 1-Step Master Mix (Life Technologies, Carlsbad, CA) with primers and probes targeting the Mhp183 gene [27], primers and probes for internal positive control (IPC) [27], and AmpliTaq® 360DNA Polymerase (5U per µL) (Thermo Fisher Scientific, Inc.). Air samples were tested using TaqMan® Fast Virus 1-Step Master Mix (Life Technologies) with primers and probes targeting the Mhp183 gene [27] and primers and probes for IPC [27]. Amplification was done on the Applied Biosystems® 7500 Fast Real-Time PCR System (ThermoFisher Scientific, Inc.). PCR results were considered valid if the IPC cycle threshold (Ct) was < 36 and samples were considered positive for *MHP* DNA when the Ct < 37.

### Clinical signs

Individual pigs were observed daily for general health. Dry, non-productive cough was measured two times per week, i.e., DPIs -3, 3, 7, 10, 14, 17, 21, 24, 31, 35, 38, 42, 45, 49, 52, 56, and 59. A single observer blinded to the pig inoculation status counted the number of coughs over a 27-min observation period (three minutes per pig) in the afternoon. At the room level, a digital quantitative cough recording system (SoundTalk®, SoundTalks NV, Leuven, Belgium) suspended in the center of each room directly over the pen counted coughs in real-time from DPI -7 through 63. The outputs provided by the system indicated the total number of coughs over a 24-h period and a respiratory distress index (RDI), which is given by a proprietary algorithm that generates warnings when the RDI is above a specified threshold [17]. The experiment was terminated on DPI 59, but the cough recording systems were left in the empty rooms to provide baseline data (DPIs 60 through 63).

### Pathologic evaluation

On DPI 59, pigs (n = 39) were humanely euthanized using captive bolt followed by exsanguination [28]. A diagnostic pathologist certified by the American College of Veterinary Pathologists and blinded to pig *MHP* status performed the necropsy and postmortem assessment. Gross lesions were scored in terms of visible lung consolidation. The score was calculated as the total percent lung consolidation by accounting for the contribution of each lobe to the total lung volume as previously described [11, 29].

At necropsy, normal and lesioned lung tissue (3 × 3 cm) were placed in 10% neutral buffered formalin and processed for histopathologic examination. Formalin-fixed tissues were embedded in paraffin wax, sectioned at 4 µm, and then stained with hematoxylin and eosin (H&E) using routine procedures. Microscopic lung lesion scores were based on BALT hyperplasia adjacent to airways and the levels of peribronchiolar infiltration by lymphocytes (PIL). Microscopic lung lesions were classified as “marked BALT hyperplasia with distortion of airway and PIL” (Score 4), “moderate BALT and PIL” (Score 3), “minimal-to-mild BALT and mild PIL” (Score 2), “minimal-to-mild BALT or PIL” (Score 1), and “no lesions” (Score 0). Scores of 3 or 4 were considered consistent with a diagnosis of porcine enzootic pneumonia [10].

### Statistical analysis

At both individual pig and room levels (Table 1), each analysis was performed based on data collected at the same time points. Analyses were performed in R (R program version 4.0.0, R core team 2020) with an alpha level ≤ 0.05 (*P* ≤ 0.05) used to establish statistical significance. The variation of PCR Ct values from *MHP* DNA positive samples over time in deep tracheal swabs, pen oral fluid, and pen drinker samples were analyzed using a linear mixed regression model and Tukey–Kramer adjustment for pairwise comparisons (*lme4* and *emmeans* R packages). To account for temporal changes in cough over the course of *MHP* infection (initiation followed by resolution), the DPI was divided into four time periods of 15 days each (DPI < 15, 15 ≤ DPI < 30, 30 ≤ DPI < 45, and DPI ≥ 45). The PCR Ct was considered as the dependent variable, time period as an independent variable, and pig (tracheal swab analysis) or room (oral fluid and drinker sample analyses) as a random effect. Comparison of quantitative PCR results from *MHP* DNA positive samples across aggregated specimens (oral fluid, drinker, and air samples) was done using linear regression model and Tukey–Kramer adjustment for pairwise comparisons (*emmeans* R packages). PCR Ct was considered the dependent variable and specimen the independent variable.

Differences in the rate of cough over time measured by the blind observer or digital recording system by rooms were analyzed using negative binomial regression with Tukey–Kramer adjustment for pairwise comparisons (*emmeans* R package). For both models, the total number of coughs was considered the dependent variable, room, DPI, and their interaction as independent variables. Spearman’s rank coefficient was used to measure the correlation between coughs measured by the blind observer and digital recording system. As the digital recording system commonly spiked on tracheal swab sampling days, days on which sampling took place were excluded from the data set. Additionally, the data collected by the system the day before sampling was used to calculate the correlation between the blind observer and the digital recording system.

The predictive value of cough for *MHP* DNA detection in aggregated specimens was estimated using four mixed-logistic models (Model A–D). The *MHP* PCR binary response (positive or negative) from pen oral fluid (Model A), pen drinker samples (Model B), and pen air sample (Model C) was considered as the dependent variable, and the frequency of coughing pigs (number of coughing pigs / total pig in a pen by DPI) in the room (Model A–C), or documented cough data by digital system (Model D) as the independent variable, and room as the random effect. In these models, DPI was added as a covariate and then the effect of cough on the *MHP* DNA detection in different specimens was measured.

The association between time period of *MHP* infection (four intervals of 15 days each, i.e., DPI < 15, 15 ≤ DPI < 30, 30 ≤ DPI < 45, and DPI ≥ 45) and score for gross lung lesions (percent consolidation, log transformed) and microscopic lung scores (BALT and PIL) were evaluated using linear regression model with Bonferroni adjustment for pairwise comparisons (*emmeans* R package) and Kruskal–Wallis test and Dunn test for pairwise comparisons (*FSA* R package), respectively. Association between total number of coughs per pig (counted after DPI 45) with gross lung scores was done using linear mixed regression, using the natural logarithm of gross lung score as dependent variable, number of coughs per pig as the independent, and the period of *MHP* infection (four intervals of 15 days each) as random effect.

Normality and homoscedasticity assumptions of linear and linear mixed regression models were analyzed using Q-Q and residuals versus fitted plots (*ggResidpanel* R package), and model fitness following categorization of continuous variables (e.g. DPI divided into four time period) was evaluated based on Akaike information criteria. Over-dispersion and deviance assumptions of negative binomial regression were verified using simulated residuals versus fitted plots (R package *DHARMa; P* > 0.05). For logistic regressions, plots (logit versus continuous were used to evaluate the linear relationship between the logit of the outcome and continuous predictors, and model fitness was evaluated using Akaike information criteria and Hosmer–Lemeshow Test (*P* > 0.05).

## Results

Pigs (n = 3) from Room 5 (negative control) were clinically normal throughout the study. One pig in Room 1 died acutely on DPI 10 and was diagnosed with chronic, fibrosing pericarditis and epicarditis. One pig in Room 4 became lame (DPI 28), was isolated and treated with ceftiofur (5.0 mg per kg; Zoetis Inc.), dexamethasone (2.0 mg per kg; MWI® Animal Health), and flunixin meglumine (2.2 mg per kg; Prevail™, MWI® Animal Health), but did not recover and was humanely euthanized on DPI 35.

*MHP* DNA detection in expected negative samples.

In Room 5 (negative control), all collected deep tracheal swabs (n = 39), pen-based oral fluid (n = 64), drinker wipes (n = 9), and air samples (n = 9) tested *MHP* PCR negative throughout the study. In Rooms 1–4, deep tracheal swabs (n = 9 per room), pen-based oral fluid (n = 5 per room), drinker wipes (n = 1 per room), and air samples (n = 1 per room) collected between DPI -4 through 0 were negative for *MHP* by PCR.

### *MHP* DNA detection in deep tracheal swab samples

**Fig. 1:**
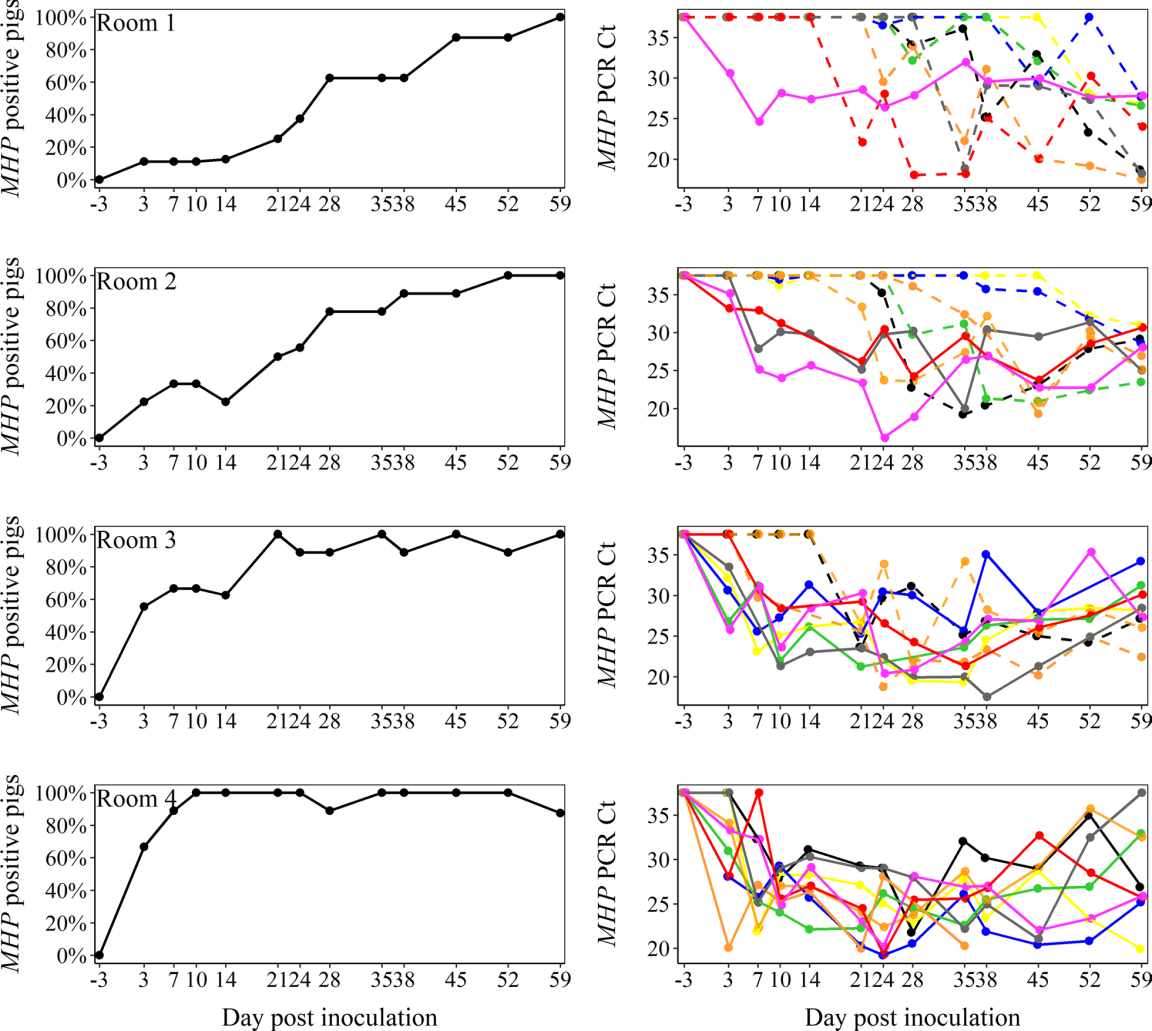
Individual pig *Mycoplasma hyopneumoniae* (*MHP*) PCR results using deep tracheal swabs over time by room. Solid or dashed colored lines represent each *MHP*-inoculated or uninoculated pig in a room, respectively. Room 1: One *MHP*-inoculated pig commingled with 8 uninoculated pigs. Room 2: 3 *MHP*-inoculated pigs with 6 uninoculated pigs. Room 3: 6 *MHP*-inoculated pigs with 3 uninoculated pigs. Room 4: 9 *MHP*-inoculated pigs

PCR results of deep tracheal swab samples by pig and room are presented in Fig. 1 and Table 2, respectively. Among deep tracheal swabs collected from inoculated pigs in Rooms 1–4, 15 of 19 *MHP*-inoculated pigs tested PCR positive on DPI 3, i.e., the one pig inoculated in Room 1, two of three pigs in Room 2, five of six pigs in Room 3, and seven of nine pigs in Room 4. The remaining inoculated pigs became *MHP* PCR positive via deep tracheal swab on DPI 7. Within-pen *MHP* transmission from pen inoculated pigs was observed through *MHP* DNA detection in deep tracheal swabs collected from uninoculated pigs in room groups. In Room 1 (1 *MHP*-inoculated: 8 uninoculated), the first *MHP* transmission event was observed in one pig on DPI 21, two pigs on DPI 24, two pigs on DPI 28, one pig on DPI 35, and the remaining pig on DPI 52, i.e., all uninoculated pigs except one pig that died on DPI 10 became *MHP* infected. In Room 2 (3 *MHP*-inoculated: 6 uninoculated), the first *MHP* transmission was observed in two pigs on DPI 10, one pig on DPI 21, one pig on DPI 24, and two pigs on DPI 28. In Room 3 (6 *MHP*-inoculated: 3 uninoculated), the first *MHP* transmission was observed in one pig on DPI 7 and the two remaining pigs on DPI 21.

**Table 2:** *Mycoplasma hyopneumoniae* (*MHP*) DNA detection by specimen and room

Specimen	*MHP* DNA detection	Room			
		1	2	3	4
		1 *MHP*-inoculated pig: 8 uninoculated pigs	3 *MHP*-inoculated pigs: 6 uninoculated pigs	6 *MHP*-inoculated pigs: 3 uninoculated pigs	9 *MHP*-inoculated pigs
Tracheal swab	First detection	DPI 3	DPI 3	DPI 3	DPI 3
	Pig-to-pig transmission detected^a^	DPI 21	DPI 10	DPI 7	NA^b^
	100% detection in group	DPI 52	DPI 38	DPI 21	DPI 7
	*MHP* PCR positive of total samples	47 of 99	69 of 107	94 of 104	98 of 103
	*MHP* PCR overall mean ± se Ct^c^	26.8 ± 0.7	27.6 ± 0.6	26.2 ± 0.4	26.2 ± 0.4
	Highest *MHP* DNA concentration (DPI)	23.4 (DPI 59)	24.4 (DPI 45)	23.6 (DPI 28)	24.3 (DPI 28)
Oral fluid	First detection	DPI 8	DPI 9	DPI 14	DPI 8
	*MHP* PCR positive of total samples^d^	17 of 59^1^	32 of 59^2^	43 of 59^2^	31 of 59^2^
	*MHP* PCR overall mean ± se Ct	34.7 ± 0.3	33.3 ± 0.5	33.5 ± 0.3	33.4 ± 0.4
	*MHP* PCR lowest Ct	31.6 (DPI 51)	25.7 (DPI 13)	27.4 (DPI 45)	26.1 (DPI 28)
Drinker	First detection	DPI 21	DPI 21	DPI 21	DPI 21
	*MHP* PCR positive of total samples	5 of 8	4 of 8	4 of 8	5 of 8
	*MHP* PCR overall mean ± se Ct	35.3 ± 1.5	33.7 ± 1.9	34.7 ± 1.4	34.7 ± 1.6
	*MHP* PCR lowest Ct	34.5 (DPI 59)	31.5 (DPI 21)	33.3 (DPI 35)	32.2 (DPI 24)
Air	First detection	ND^e^	DPI 10	ND^e^	DPI 17
	*MHP* PCR positive of total samples	ND^e^	5 of 26	ND^e^	3 of 26
	*MHP* PCR overall mean ± se Ct		36.2 ± 0.4		36.3 ± 0.6

^a^First pig-to-pig transmission based on detection of *MHP* DNA in deep tracheal swab from commingled animal

^b^NA: not applicable

^c^Negative values (Ct ≥ 37) were excluded from calculations of statistical descriptive measures

^d^Different superscripted numbers (1, 2, 3, and 4) indicate statistical differences (*P* ≤ 0.05, logistic regression and Tukey–Kramer pairwise comparisons)

^e^ND: not done

*MHP* DNA was consistently detected following first detection until the end of the study in deep tracheal swabs from inoculated pigs, with a total of 211 PCR positive deep tracheal swabs of 218 (97%). Following the first positive PCR result, *MHP* DNA was consistently detected until the end of the study in deep tracheal swabs in all three uninoculated pigs from Room 3. Within Room 1 and 2, all eight or six uninoculated pigs, respectively, were consistently PCR positive over the study, with exception of two pigs in each room. From those four pigs, in two or three time points, deep tracheal swabs resulted in *MHP* DNA negative following the first positive PCR result. A total of 97 positive PCR results of 195 collected deep tracheal swabs (50%) was observed in uninoculated pigs. The mean distribution of Ct values from *MHP* PCR positive tracheal swabs by room throughout the study is given in Table 2. Comparisons of individual pig tracheal swab PCR Ct values (least square means) by time period showed lower *MHP* DNA concentrations (*P* < 0.004, linear mixed model) at DPI < 15 (28.3; 95 CI% 27.1, 29.4) and DPI ≥ 45 (26.7; 95 CI% 25.7, 27.7) as compared to middle periods, i.e., 15 ≤ DPI < 30 (26.0; 95 CI% 24.8, 27.1), 30 ≤ DPI < 45 (26.3; 95 CI% 25.1, 27.2).

### *MHP* DNA detection in pen oral fluid samples

After inoculation, 59 pen-based oral fluid samples were collected from each *MHP*-inoculated Room from DPI 1 through 59 (n = 236 samples). As shown in Table 2, *MHP* DNA was first detected in oral fluid samples from Rooms 1 and 4 on DPI 8, Room 2 on DPI 9, and Room 3 on DPI 14. Throughout the study, *MHP* DNA was detected in 17 of 59 pen oral fluids (29%) in Room 1, 32 of 59 (54%) in Room 2, 43 of 59 (73%) in Room 3, and 31 of 59 (53%) in Room 4. The rate of PCR positive oral fluid samples was lower in Room 1 vs Rooms 2, 3, and 4 (*P* < 0.001, logistic regression). The mean distribution of Ct values from *MHP* PCR positive oral fluids by room throughout the study is given in Table 2. Within room, there was no significant variation in oral fluid PCR Ct values (least square means) by time period (*P* > 0.05, linear mixed model).

### *MHP* DNA detection in pen drinker samples

Following inoculation, eight drinker samples per *MHP*-inoculated room (n = 32 samples) were collected on DPIs 7, 14, 21, 24, 35, 45, 52, and 59. On DPI 21, *MHP* DNA was detected in all drinker samples. *MHP* DNA was detected in five of eight pen drinker samples (62.5%) in Room 1 and 4, and four of eight pen drinker samples (50%) in Rooms 2 and 3. The mean distribution of Ct values from *MHP* PCR positive drinker samples by room is given in Table 2. Within room, there was no significant variation in pen drinker PCR Ct values (least square means) by period (*P* > 0.05, linear mixed model).

### *MHP* DNA detection in pen air samples

Air samples were collected three times per week from Rooms 2 and 4 (*MHP*-inoculated rooms), i.e., a total of 26 samples from each room following inoculation (2, 3, 7, 9, 10, 14, 16, 17, 21, 23, 24, 28, 30, 31, 35, 37, 38, 42, 44, 45, 49, 51, 52, 56, 58, and 59 DPIs). Among these, *MHP* DNA was detected in Room 2 air samples on DPIs 10, 14, 51, 52, and 59 and Room 4 on DPIs 17, 31, and 42. Throughout the study, the distribution of Ct values from *MHP* PCR positive pen air samples was 36.2 (standard error = 0.4) in Room 2, and 36.3 (standard error = 0.6) in Room 4 (Table 2). Due to the low number of positive PCR results for *MHP* DNA in air samples, the analysis of distribution of PCR Cts over DPIs was not done. No statistical difference among PCR Ct values from aggregated samples (pen oral fluids, pen drinker wipes, and pen air samples) was observed (*P* > 0.05, linear regression model).

### Cough data based on four scenarios of within-pen *MHP* prevalence

**Fig. 2:**
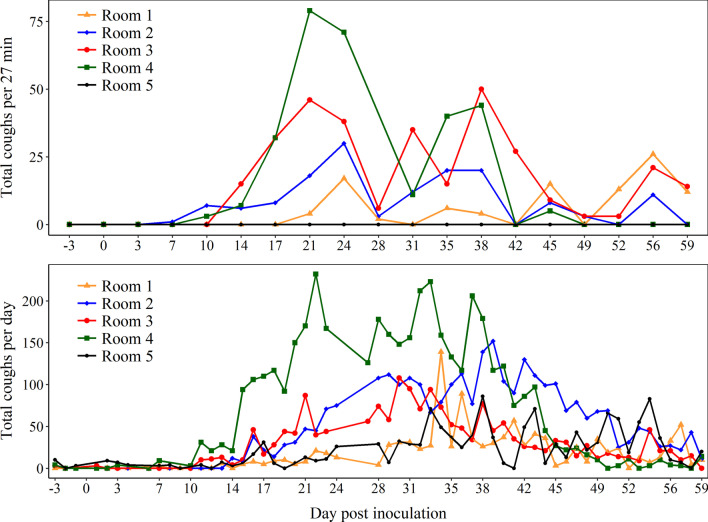
Pattern of coughs counted based on a blinded observer to pig *Mycoplasma hyopneumoniae (MHP)*-inoculation status (27-min period) and digital recording system by rooms (24-h period, SoundTalk®, SoundTalks NV, Leuven, Belgium). Room 1: One *MHP*-inoculated pig commingled with 8 uninoculated pigs. Room 2: 3 *MHP*-inoculated pigs with 6 uninoculated pigs. Room 3: 6 *MHP*-inoculated pigs with 3 uninoculated pigs. Room 4: 9 *MHP*-inoculated pigs. Room 5: 3 uninoculated pigs

Cough data per the observer or digital recording system by room are shown in Fig. 2 and Table 3. In Rooms 1–4, dry, non-productive coughs were first noted by the observer on DPI 10 (Rooms 2 and 4), DPI 14 (Room 3) and DPI 21 (Room 1). Thereafter, cough was continuously observed until the termination of the study in Rooms 1, 2, and 3, but not after DPI 45 in Room 4. The coughs rate was significantly higher in Rooms 3 and 4 compared to 1 and 2 (*P* < 0.001, negative binomial regression, Table 3). No dry, non-productive cough was observed in Room 5 (negative control).

**Table 3:** Cough indices and pathologic lesions associated with *Mycoplasma hyopneumoniae* (*MHP*) infection by room

Clinical assessment and pathology	Room^a^				
	1	2	3	4	5
Cough counted per pig over 27-min (blind observer to *MHP* status)^b^					
First observation	DPI 21	DPI 10	DPI 14	DPI 10	-
Coughs per 27 min (mean ± se)	5.8 ± 1.9	8.7 ± 2.1	18.5 ± 4.1	18.3 ± 6.7	-
Coughing pigs per day (mean ± se)	1.9 ± 0.8	1.8 ± 1.0	3.8 ± 2.0	4.6 ± 2.5	0
Number of days with coughing pigs	9	13	14	9	0
Total number of coughs	99^1^	147^1^	314^2^	292^2^	0
Maximum cough (number of coughs)	DPI 56 (26)	DPI 24 (30)	DPI 38 (50)	DPI 21 (79)	-
Coughs recorded over 24-h (SoundTalk®, SoundTalks NV, Leuven, Belgium)^c^					
Coughs per 24 h (mean ± se)	23.4 ± 4.1	55.6 ± 6.6	33.1 ± 4.1	76.6 ± 11.2	23.1 ± 3.6
Total number of coughs	959^1^	2,279^2,3^	1,390^1,2^	3,292^3^	992^1^
Maximum cough (number of coughs)	DPI 34 (139)	DPI 39 (152)	DPI 30 (108)	DPI 22 (232)	DPI 54 (83)
Respiratory distress index (mean ± se)	0.02 ± 0.01	0.17 ± 0.03	0.04 ± 0.01	0.12 ± 0.02	0.01 ± 0.01
Maximum respiratory distress index	DPI 34 (0.20)	DPI 31 (0.58)	DPI 30 (0.22)	DPI 35 (0.53)	DPI 3 (0.41)
Lung score					
Median gross lesion (min–max)	2.9% (0.6–36)	1.1% (0–12)	1.1% (0–18)	0.4% (0–3.8)	0% (0–0)
Median microscopic score (min–max)	4 (3–4)	3 (1–4)	4 (1–4)	3 (2–4)	1 (1–1)

^a^Room 1: One *MHP*-inoculated pig commingled with 8 uninoculated pigs. Room 2: 3 *MHP*-inoculated pigs with 6 uninoculated pigs. Room 3: 6 *MHP*-inoculated pigs with 3 uninoculated pigs. Room 4: 9 *MHP*-inoculated pigs. Room 5: 3 uninoculated pigs

^b^Different superscripted numbers (1 or 2) indicate statistical differences among Room groups (*P* ≤ 0.05, negative binomial regression and Tukey–Kramer pairwise comparisons)

^c^Different superscripted numbers (1, 2, and 3) indicate statistical differences among Room groups (*P* ≤ 0.05, negative binomial regression and Tukey–Kramer pairwise comparisons)

The digital recording system documented coughs and RDIs at various time points between -4 and 59 DPIs (n = 64 time points) in all Rooms (Fig. 2; Table 3). However, no warnings due to respiratory distress were generated. In Room 2, no RDI data was provided by the algorithm between DPIs 35 through 63 because of an internet connection issue. The first cough event in Rooms 3, 4 and 5 was noted at -3 DPI (the first day of tracheal sampling) and at 7 and 14 DPI for Rooms 1 and 2, respectively. The maximum number of coughs recorded by the system ranged from 83 for Room 5 at 54 DPI to 232 for Room 4 at 22 DPI. The cough rate documented by the digital recording system was statistical higher and similar in Rooms 4 and 2, and no statistically significant difference was detected among Rooms 1, 3, and 5 (*P* < 0.001, negative binomial regression). A moderate association (0.58; *P* < 0.001) was observed between the total number of coughs in a given DPI recorded by the blind observer and the digital system.

### Predictive cough for *MHP* DNA in aggregated specimens

**Fig. 3:**
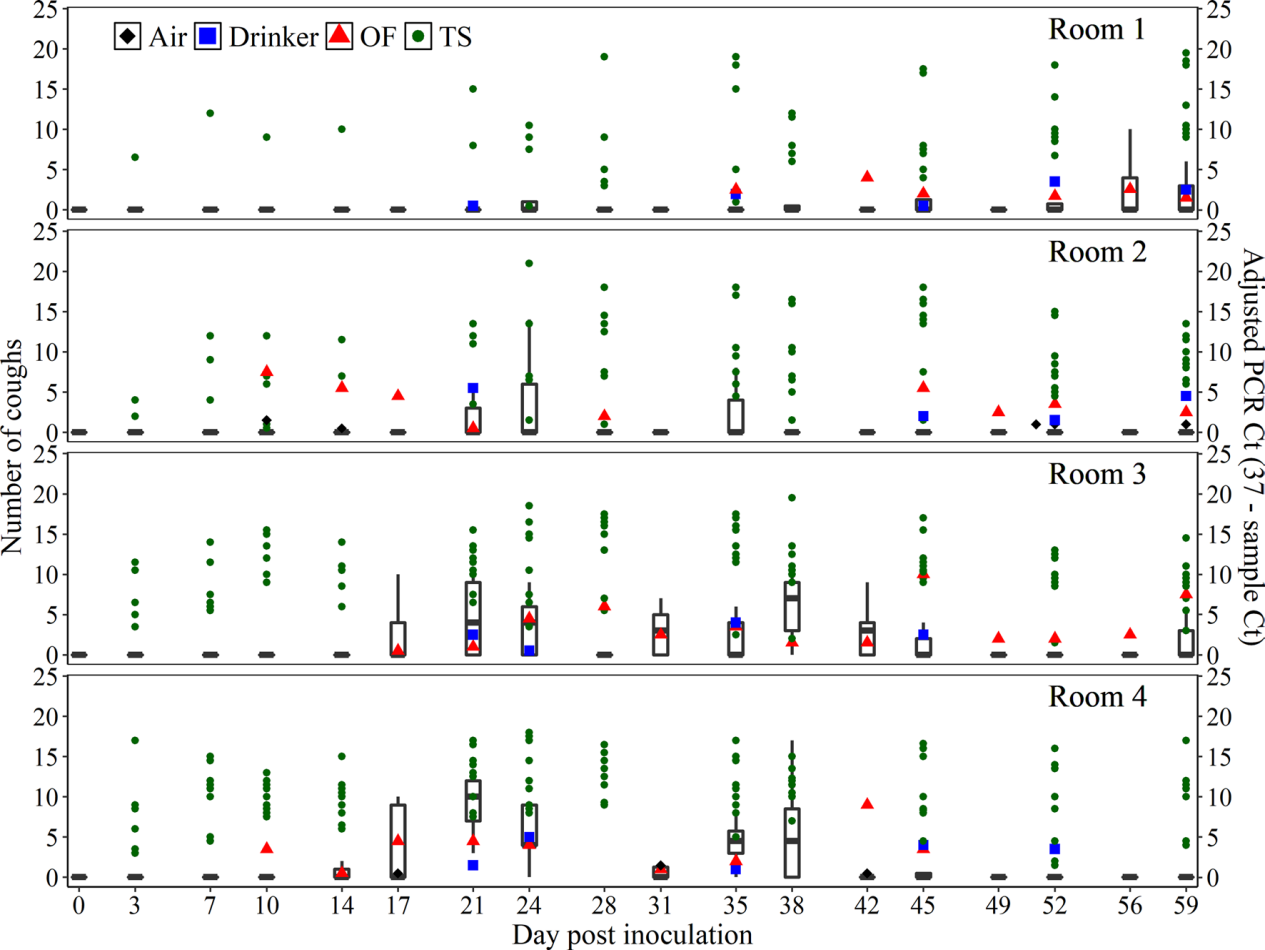
Number of coughs (box plot with median and interquartile range) related to *Mycoplasma hyopneumoniae (MHP)* DNA concentration (Adjusted PCR Ct) from deep tracheal swabs (TS; dark-green dots represent a pig), pen oral fluid (OF; red triangles represent a pen in a room), pen drinker (blue squares represent a pen in a room), and pen air samples (black diamonds represent a pen in a room). Room 1: One *MHP*-inoculated pig commingled with 8 uninoculated pigs. Room 2: 3 *MHP*-inoculated pigs with 6 uninoculated pigs. Room 3: 6 *MHP*-inoculated pigs with 3 uninoculated pigs. Room 4: 9 *MHP*-inoculated pigs

**Fig. 4:**
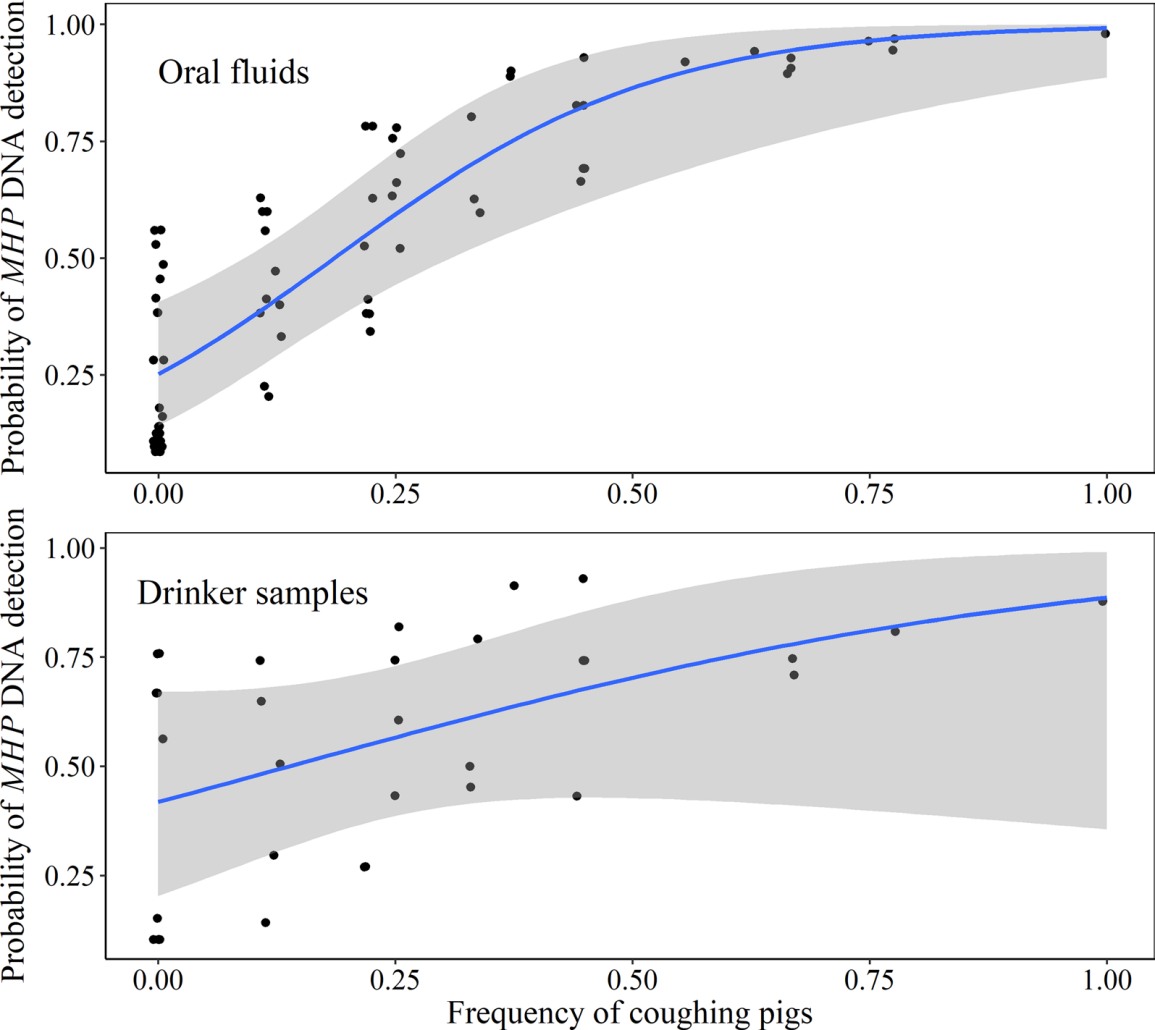
Overall probability of detection *Mycoplasma hyopneumoniae* DNA in oral fluid and drinker samples as a function of frequency of coughing pigs in a pen

Figure 3 shows the cough pattern measured by the observer related to *MHP* DNA concentration by specimen. The frequency of coughing pigs, e.g., the number of coughing pigs divided by total number of pigs in pen given a DPI, as measured by the observer was a significant predictor for *MHP* DNA detection in pen oral fluid samples (beta coefficient = 4.85; SE = 1.59; *P* < 0.001; Model A) and nearly significant in pen drinker wipes (beta coefficient = 3.25; SE = 1.88; *P* = 0.08; Model B). As shown in Fig. 4, the probability of detecting *MHP* DNA in pen oral fluid and drinker samples increased as the frequency of coughing pigs increased. Likewise, the probability of *MHP* DNA detection increased by 1% as the cough data generated by the digital system increased by 1-unit (beta coefficient = 0.01; SE = 0.001; *P* < 0.001; Model D). The value of cough as a predictor for *MHP* DNA detection in air samples (Model C) was not done because of the limited number of positive PCR results in this study.

### Pathologic evaluation

Gross and microscopic lung lesions are given in Table 3. Gross lung lesions at necropsy were significantly higher in pigs that became infected ≥ DPI 15 versus those infected < DPI 15 (*P* < 0.002, linear regression). Microscopic lung scores did not differ between *MHP*-infected pigs by time period (*P* ≥ 0.05, Kruskal–Wallis test). The percent of lung consolidation was positively associated with total number of coughs per pig counted after DPI 45 (*P* < 0.001, linear mixed regression). That is, the median percent of lung consolidation increased when the number of coughs per pig increased by 7.6% (95 CI% 5.2%, 10.5%).

## Discussion

The objective of this study was to evaluate the value of cough as a predictor for *MHP* DNA detection in different aggregated specimens. Cough due to *MHP* infection is triggered by the accumulated mucus and partial compression of airways by BALT hyperplasia [9–11]. The internal validity of the study was supported by the detection of *MHP* by PCR in tracheal swab samples from all pigs within *MHP*-positive rooms, gross and histologic lung lesions consistent with *MHP* disease, and the positive association between the percent of lung consolidation and total number of coughs per pig counted after DPI 45. In this study, microscopic lung score was not associated with time of *MHP* infection, but only a single section of lung was evaluated.

There was a significant association between the frequency of coughing pigs in a pen and *MHP* DNA detection in oral fluids, suggesting that pen-based oral fluid samples are an appropriate diagnostic specimen to evaluate for the presence of *MHP* by PCR in the presence of cough under the conditions of this study. Given that *MHP* is more likely to be found in the lower respiratory tract [15], cough may contribute to the presence of *MHP* in the oral cavity, increasing the likelihood of detecting *MHP* DNA in oral fluid samples. This has been suggested but not evaluated by Clavijo et al. [17] and Hernandez-Garcia et al. [22].

Pen drinker samples are not a common sample type submitted for molecular diagnostics. However, design of drinkers in modern production systems effectively flushes the oral cavity of each animal as it consumes water, providing a continuous, autonomous (labor-less), aggregated, non-invasive, population-based sample that could be assessed for various pathogens. In this study, analyses showed that PCR Ct values of drinker samples did not differ from the Ct values of oral fluid samples, but the model of cough as a predictor for *MHP* DNA detection in drinker samples was not significant (*P* = 0.08). This may have been impacted by the small sample size and limited sampling frequency in this study. The rate of detection of nucleic acid by PCR of water samples may be affected by PCR inhibitors [30]. In this study, PCR inhibitors in the water due to sample processing or nucleic acid extraction were monitored by the use of an internal positive control in every sample at the DNA extraction step [27]. The usefulness of pen drinker samples likely warrants further investigation.

In this study, air sampling resulted in occasional *MHP* PCR positive samples. Poeta Silva et al. did not detect *MHP* by PCR or culture using a similar cyclonic liquid impinger [26]. However, the duration of sampling (60 min versus 15 min), location of the air sampler (inside versus outside the pen) and PCR protocol differed. Under field conditions, Dee et al. [31], rarely (5%) detected *MHP* DNA by PCR near the exhaust fan in an *MHP*-positive herd using a cyclonic liquid impinger. These findings suggest that while detection of *MHP* in air samples is inconsistent that identification of *MHP* nucleic acid in this sample type could indicate numerous pigs are infected with *MHP*.

Due to the colonization site of *MHP* [17–19], *MHP* DNA was detected earliest in deep tracheal swabs, e.g., 15 of 19 *MHP*-inoculated pigs on DPI 3 with all commingled, non-inoculated pigs becoming positive at some point in the study (Table 2). Roos et al. [32] showed that a 100% within pen *MHP* infection prevalence was achieved with a ratio of six *MHP*-inoculated and four uninoculated pigs by DPI 28. While, in this study, a 100% within-pen *MHP* infection prevalence, based on *MHP* DNA detection in deep tracheal swabs, was achieved with a ratio of six *MHP*-inoculated and three uninoculated pigs on DPI 21 and three *MHP*-inoculated pigs and six uninoculated on DPI 38. That is, not surprisingly, transmission events based on *MHP* DNA detection by PCR via tracheal swabs occurred more quickly in pens with more *MHP*-infected pigs. As a general trend, *MHP* DNA concentration in tracheal swabs was lower through DPI 15, higher and steady from DPI 15 to 45, and lower after DPI 45, thus suggesting that the rate of *MHP* replication varied over time.

Analyses of the cough pattern measured by the observer demonstrated that cough rate was positively associated with within-pen *MHP* prevalence after DPI 15, as demonstrated by the higher cough observed in Room 3 (6 of 9 *MHP*-inoculated pigs) and 4 (9 of 9 *MHP*-inoculated pigs) (Fig. 2). Likewise, a higher cough incidence rate was observed in Rooms 2 and 4 using the digital recording system; however, there was no statistical difference among Rooms 1, 3, and 5 (negative control group). There was moderate correlation between cough measured by the observer and digital recording system. This moderate rather than strong correlation may be a result of multiple factors. First, it may reflect the difference in time used by each methodology (27 min vs 24 h). Second, based on the manufacturer’s instructions, these devices were designed to capture specific cough sounds in larger pig populations [33]. Therefore, in this study, the limited number of pigs in the room and/or the acoustics of the BSL-2 facility may have led to the absence of warnings and lower values of RDI by the digital recording system. This might have limited the use of the digital recording system under the conditions of this study, suggesting that the algorithm needs to be adapted to controlled environment. Clavijo et al. [17] reported at least seven warnings in a commercial wean-to-finish farm (1,250 pigs per room) undergoing *MHP* infection. Thus, their use under field conditions has been supported for *MHP* surveillance in larger pig populations.

## Conclusion

The selection of specimens and testing will depend on the objectives of *MHP* surveillance. The data from this study suggests that in the presence of cough and high within-pen *MHP* prevalence, oral fluids and drinker samples may provide a more convenient (aggregate sampling with no pig handling) means to surveil the population for *MHP* infection than the more laborious tracheal sampling. Thus, these specimens may be utilized in herds with suggestive clinical signs of *MHP* infection.

## Data Availability

The datasets during and/or analyzed during the current study available from the corresponding author on reasonable request.

## Abbreviations

BALT: Bronchus-associated lymphoid tissues
DNA: Deoxyribonucleic acid
DPI: Day post inoculation
ELISA: Enzyme-linked immunosorbent assay
H&E: Hematoxylin and eosin
IAV: Influenza A virus
ISU VDL: Iowa State University Veterinary Diagnostic Laboratory
*MHP*: *Mycoplasma hyopneumoniae*
PCR: Polymerase chain reaction
PCV2: Porcine circovirus 2
PEP: Porcine enzootic pneumonia
PIL: Peribronchiolar infiltration by lymphocytes
PRRSV: Porcine reproductive and respiratory syndrome virus
